# Kinesin family member 18B activates mTORC1 signaling via actin gamma 1 to promote the recurrence of human hepatocellular carcinoma

**DOI:** 10.1038/s41389-023-00499-7

**Published:** 2023-11-13

**Authors:** Qian Li, Mengqing Sun, Yao Meng, Mengqing Feng, Menglan Wang, Cunjie Chang, Heng Dong, Fangtian Bu, Chao Xu, Jing Liu, Qi Ling, Yiting Qiao, Jianxiang Chen

**Affiliations:** 1 School of Pharmacy and Department of Hepatology, the Affiliated Hospital of Hangzhou Normal University, Hangzhou Normal University, Hangzhou, 311121 P. R. China; 2 Key Laboratory of Elemene Class Anti-Cancer Chinese Medicines; Engineering Laboratory of Development and Application of Traditional Chinese Medicines; Collaborative Innovation Center of Traditional Chinese Medicines of Zhejiang Province, Hangzhou Normal University, Hangzhou, Zhejiang 311121 P. R. China; 3 Division of Hepatobiliary and Pancreatic Surgery, Department of Surgery, NHC Key Laboratory of Combined Multi-organ Transplantation, Key Laboratory of Organ Transplantation, Research Center for Diagnosis and Treatment of Hepatobiliary Diseases, The First Affiliated Hospital, Zhejiang University School of Medicine, Hangzhou, Zhejiang 310003 P. R. China; 4 Jinan Microecological Biomedicine Shandong Laboratory, Jinan, Shandong 250000 P. R. China; 5 Laboratory of Cancer Genomics, Division of Cellular and Molecular Research, National Cancer Centre, Singapore, 169610 Singapore

**Keywords:** Gastrointestinal cancer, Cell signalling

## Abstract

The mechanistic target of rapamycin complex 1 (mTORC1) signaling pathway is frequently reported to be hyperactivated in hepatocellular carcinoma (HCC) and contributes to HCC recurrence. However, the underlying regulatory mechanisms of mTORC1 signaling in HCC are not fully understood. In the present study, we found that the expression of kinesin family member 18B (KIF18B) was positively correlated with mTORC1 signaling in HCC, and the upregulation of KIF18B and p-mTOR was associated with a poor prognosis and HCC recurrence. Utilizing in vitro and in vivo assays, we showed that KIF18B promoted HCC cell proliferation and migration through activating mTORC1 signaling. Mechanistically, we identified Actin gamma 1 (γ-Actin) as a binding partner of KIF18B. KIF18B and γ-Actin synergistically modulated lysosome positioning, promoted mTORC1 translocation to lysosome membrane, and prohibited p70 S6K from entering lysosomes for degradation, which finally led to the enhancement of mTORC1 signaling transduction. Moreover, we found that KIF18B was a direct target of Forkhead box M1, which explains the potential mechanism of KIF18B overexpression in HCC. Our study highlights the potential of KIF18B as a therapeutic target for the treatment of HCC.

## Introduction

Primary liver cancer is the fourth leading cause of cancer-related death worldwide, with estimated 906,000 new cases and 830,000 deaths in 2020 [1]. Hepatocellular carcinoma (HCC) accounts for the majority of primary liver cancer, integrated studies have demonstrated that HCC is highly heterogeneous, with alternations in genetics, molecules, and cell signaling pathways among patients and even among different compartments within one tumor [2, 3]. Although considerable progress has been achieved in previous studies [4, 5], the detailed molecular mechanisms of hepatocarcinogenesis still remain obscure. On the other hand, despite the clinical management of HCC has been improved over the past decades, the prognosis of HCC patients is still dismal, with a 5-year survival of 18% [6]. Surgical resection and liver transplantation are the most commonly used curative managements of HCC, however, both managements share a high rate of recurrence and ultimately lead to death [7]. Therefore, it is urgently needed to reveal the underlying molecular mechanisms of HCC recurrence for the purpose of identifying novel therapeutic targets.

The mechanistic target of rapamycin (mTOR) signaling pathway integrates environmental inputs, including nutrients, stresses, oxygen and growth factors, to control eukaryotic cell metabolism and growth [8]. The mTOR is an atypical serine/threonine protein kinase that belongs to the phosphoinositide 3-kinase (PI3K)-related kinase family and interacts with several proteins to form 2 distinct complexes, named mTOR complex 1 (mTORC1) and mTOR complex 2 (mTORC2). mTORC1 must translocate to the lysosomal surface to be activated by the small GTPase Rheb [9]. Once activated, mTORC1 phosphorylates the eukaryotic initiation factor 4E binding protein 1 (4EBP1) and p70 S6 kinase 1 (p70 S6K), and ultimately promotes protein synthesis [10, 11]. In HCC, the mTOR signaling pathway is frequently upregulated as compared with matched normal liver tissues and contributes to HCC early recurrence [12, 13]. However, the underlying regulatory mechanisms of mTOR signaling in HCC are not fully understood.

Kinesin family member 18B (KIF18B) is a member of kinesin-8 family containing a plus-end-directed N-terminal motor domain [14]. KIF18B can depolymerize microtubules or cap the microtubule plus ends, thus controlling microtubule length, spindle assembly and chromosome alignment [15–19]. Numerous studies have shown that KIF18B may act as an oncogene in solid tumors, such as colon adenocarcinoma, cervical cancer, osteosarcoma, lung adenocarcinoma, pancreatic ductal adenocarcinoma and HCC [20–25]. Recently, KIF18B has been reported to promote HCC development and progression via activating Wnt/β-catenin signaling pathway, but the detailed mechanism remains unclear [22].

In this study, we showed that the expression of KIF18B was correlated with mTORC1 signaling in HCC tissues and KIF18B played an oncogenic role in HCC recurrence by enhancing mTORC1 signaling. Mechanistically, we demonstrated that KIF18B interacted with Actin gamma 1 (γ-Actin) to regulate lysosome positioning, thus promoting mTOR translocation to lysosomal surface and preventing p70 S6K from entering lysosomes for degradation. Moreover, we found that KIF18B was a direct target of Forkhead box M1 (FOXM1), which explained the mechanism of KIF18B overexpression in HCC. Our study elucidates the oncogenic role of KIF18B and highlights its application as a potential therapeutic target for HCC.

## Results

### KIF18B is positively correlated with mTORC1 signaling in HCC tissues and associated with HCC recurrence

**Fig. 1:**
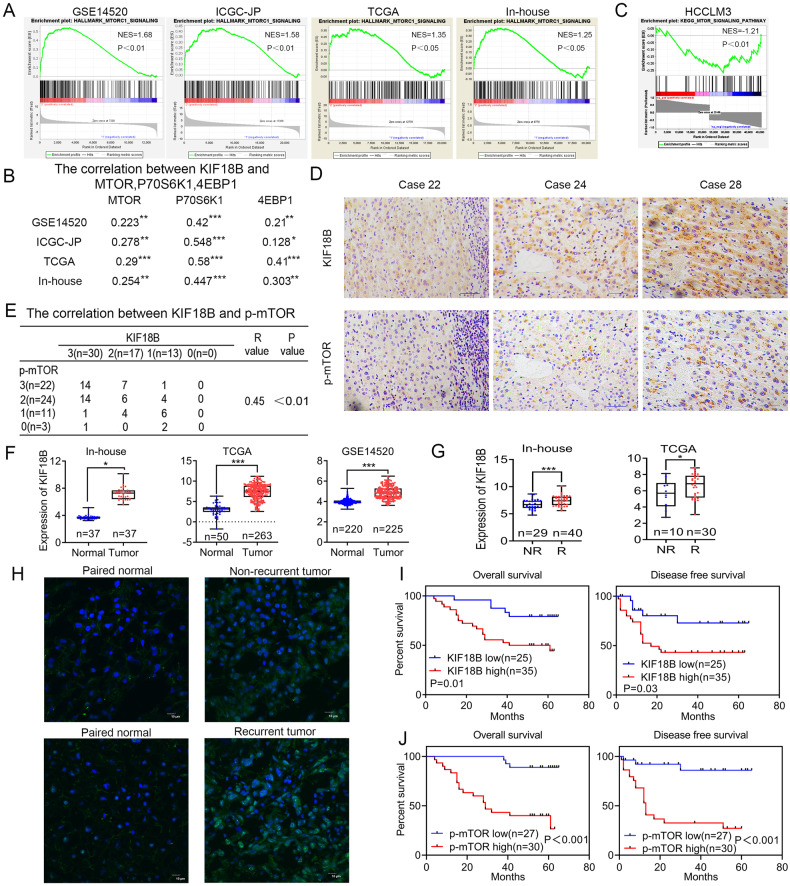
KIF18B expression is positively correlated with mTORC1 signaling in HCC and associated with HCC recurrence. **A** Gene Set Enrichment Analysis (GSEA) of microarray datasets from HCC samples in NCBI GEO (GSE14520), International Cancer Genome Consortium (ICGC-JP), The Cancer Genome Atlas (TCGA) and in-house microarray. **B** Pearson correlation analysis of KIF18B and MTOR, P70S6K1, 4EBP1 expressions in 4 datasets. **C** GSEA of the differentially expressed genes induced by KIF18B knockdown in HCCLM3 cells. **D** Immunohistochemical (IHC) analysis of KIF18B and p-mTOR expression in HCC tissues. **E** Spearman correlation analysis of KIF18B and p-mTOR expression levels in 60 HCC patients. **F** Comparison of KIF18B mRNA expression in HCC and adjacent normal tissues in in-house, TCGA, and GSE14520 datasets. **G** Comparison of KIF18B mRNA expression in non-recurrent (NR) and recurrent (R) HCC tissues in in-house and TCGA datasets. **H** Immunofluorescence detection of KIF18B in non-recurrent, recurrent HCC tissues, and corresponding normal tissues. Scale bar, 10 μm. **I** Kaplan–Meier survival analysis of overall survival (OS) or disease-free survival (DFS) according to KIF18B expression in 60 HCC patients (log-rank test). **J** Kaplan–Meier survival analysis of OS or DFS according to p-mTOR expression in 57 HCC patients (log-rank test). The error bars indicate the mean ± SD. *P* values were calculated by student *t* test, *P < 0.05, **P < 0.01, ***P < 0.001.

In order to explore the biological functions of KIF18B in HCC, we performed Gene Set Enrichment Analysis (GSEA) for microarray datasets of HCC samples in NCBI GEO (GSE14520), KIF18B expression was positively correlated with mTORC1 signaling (Fig. S1A). Interestingly, GSEA of HCC samples in International Cancer Genome Consortium (ICGC-JP), The Cancer Genome Atlas (TCGA) and in-house cohort also revealed that KIF18B expression was positively correlated with mTORC1 signaling (Fig. 1A). Moreover, the expression levels of three key components of mTORC1 signaling, MTOR, P70S6K1, and 4EBP1 were all positively correlated with KIF18B expression in these datasets (Fig. 1B). To further verify the correlation between KIF18B and mTORC1 signaling, HCCLM3 cells were transfected with siRNA specifically targeting KIF18B and were harvested for RNA sequencing. GSEA result showed that reduced KIF18B expression was significantly correlated with weakened mTORC1 signaling, thus confirming the positive correlation between KIF18B and mTORC1 signaling (Fig. 1C). To validate this correlation on protein level, we first conducted immunofluorescence (IF) to detect the localization of mTOR and KIF18B. The results showed co-expressed of KIF18B and mTOR in the tissues (Fig. S2). Then, IHC was performed to evaluate the expression levels of both KIF18B and p-mTOR in 60 HCC cases. Spearman test on IHC scores demonstrated that the protein level of KIF18B was positively correlated with the protein level of p-mTOR in clinical samples (Fig. 1D, E). Additionally, analysis of the in-house, GSE14520 and TCGA datasets showed that KIF18B mRNA was significantly higher in HCC tissues compared with normal liver tissues (Fig. 1F). Surprisingly, the mRNA and protein expressions of KIF18B were markedly upregulated in recurrent tissues compared with non-recurrent HCC tissues (Fig. 1G, H). Further analysis of the survival outcomes of HCC patients revealed that patients with relatively higher KIF18B or p-mTOR expression had poorer overall survival and disease-free survival than those with relatively lower KIF18B or p-mTOR expression (Fig. 1I, J). These results suggest that KIF18B might have a role in the hyper-activation of mTORC1 signaling and contribute to HCC progression and recurrence.

### KIF18B is required to maintain the oncogenesis of HCC cells by potentiating the proliferation and migration, and suppressing the apoptosis

**Fig. 2:**
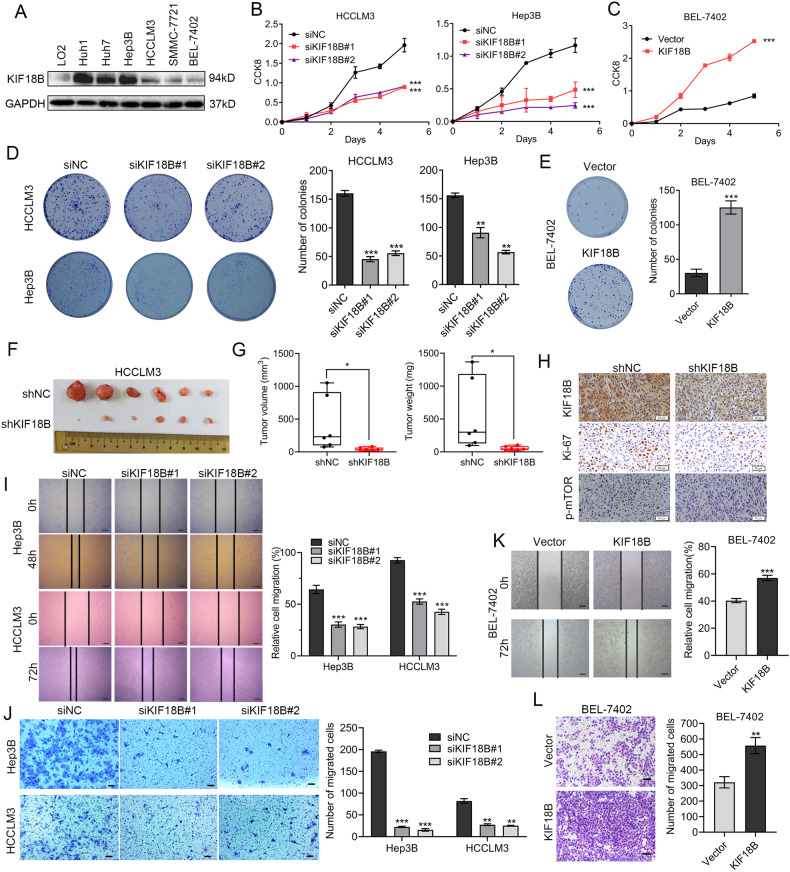
KIF18B promotes HCC cell proliferation and migration. **A** Expression of KIF18B in normal human hepatic cell line and HCC cell lines examined by western blot. **B**, **C** The influence of KIF18B silencing (**B**) and ectopic expression (**C**) on HCC cell proliferation determined by cell counting kit-8 (CCK-8) assay. **D**, **E** Colony formation images of KIF18B silencing (**D**) and KIF18B overexpression (**E**) HCC cells. The colonies were captured and counted. **F** The growth of HCC subcutaneous tumors is suppressed by KIF18B silencing. **G** Subcutaneous tumors were measured by volume and weight. **H** IHC analysis of the expression levels of KIF18B, Ki-67, and p-mTOR in the indicated subcutaneous tumors. Scale bars, 50 μm. **I**, **K** The influence of KIF18B silencing (**I**) and ectopic expression (**K**) on HCC cell migration determined by wound healing assay. Scale bars, 200 μm. **J**, **L** Transwell assay images of KIF18B silencing (**J**) and overexpression (**L**) HCC cells. The cells were captured and counted. Scale bars, 100 μm. Error bars indicate the mean ± SD of three independent experiments. *P* values were calculated by student *t* test, *P < 0.05, **P < 0.01, ***P < 0.001.

KIF18B expression was measured in 6 human HCC cell lines and normal human hepatic cell line LO2 by qRT-PCR and western blot. Consistent with the data from human tissues, both KIF18B mRNA and protein expressions were higher in HCC cell lines compared with LO2 (Fig. 2A and S1B). To characterize the biological effects of either suppression or overexpression of KIF18B in HCC cells, siRNA and lentivirus were used to either reduce or increase the expression level of KIF18B. Western blot results showed that KIF18B was effectively silenced in HCCLM3 and Hep3B cells, both of which had a relatively good level of endogenous KIF18B expression, and KIF18B was overexpressed in BEL-7402 cells in which endogenous KIF18B level was low (Fig. S1E). As shown by cell viability assays, KIF18B silencing remarkably inhibited the proliferation of HCC cells, while ectopic KIF18B expression exhibited the opposite effect (Fig. 2B, C). Consistent with these results, colony formation was attenuated by silencing KIF18B and enhanced by KIF18B overexpression (Fig. 2D, E). These results indicate that KIF18B has an oncogenic role in HCC cells in vitro.

To demonstrate the oncogenic role of KIF18B in vivo, we established xenograft tumors by subcutaneously injecting the same number of HCCLM3 cells carrying scramble shRNA or cells carrying shRNA targeting KIF18B into nude mice. As expected, KIF18B silencing significantly reduced the size and weight of HCCLM3 xenografts in vivo (Fig. 2F, G). IHC analysis of xenograft tissues also confirmed that reduced KIF18B would lead to reduced cell proliferation as indicated by the frequency of Ki-67 positive cells (Fig. 2H). Furthermore, we found that KIF18B silencing reduced the expression of p-mTOR in HCCLM3 xenografts, which was consistent with our previous observations in clinical specimens (Fig. 2H).

We then investigated whether change of KIF18B expression would affect migration and apoptosis for HCC cells. Transwell migration and wound healing assays demonstrated that KIF18B silencing suppressed the migration of Hep3B and HCCLM3 cells in vitro (Fig. 2I, J). Vice versa, the migratory ability of BEL-7402 cells significantly increased after ectopic expression of KIF18B (Fig. 2K, L). Flow cytometric analysis of Annexin V-FITC/PI indicated that KIF18B silencing stimulated apoptosis in Hep3B and HCCLM3 cells (Fig. S1C), whereas KIF18B overexpression reduced apoptosis in BEL-7402 cells (Fig. S1D). Consistently, KIF18B silencing enhanced protein levels of cleaved caspase3 and cleaved PARP in Hep3B and HCCLM3 cells, while ectopic KIF18B expression exhibited the opposite effects in BEL-7402 cells (Fig. S1E). Taken together, these data suggest that KIF18B might function as an oncogene and promote HCC progression and recurrence.

### KIF18B acts as an oncoprotein in HCC cells by enhancing mTORC1 signaling

**Fig. 3:**
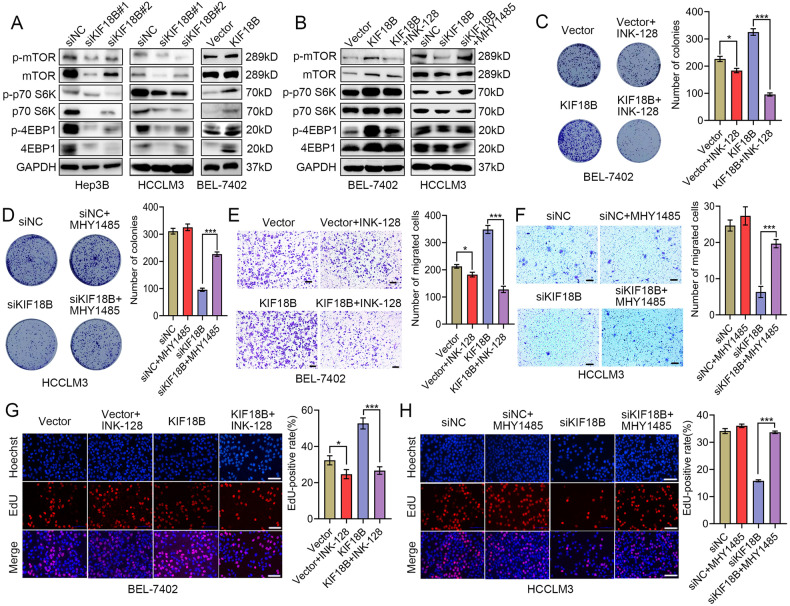
KIF18B acts as an oncoprotein in HCC cells through activating mTORC1 signaling. **A** Western blot analysis of the protein and phosphorylation levels of mTOR and its substrates (p70 S6K and 4EBP1) in the KIF18B-depleted and -overexpressed HCC cells. **B** Western blot analysis showing that MHY1485 restores the KIF18B silencing-induced phosphorylation inhibition of mTOR, p70 S6K, and 4EBP1. While, INK-128 treatment eliminates the activity of mTORC1 signaling enhanced by KIF18B overexpression. **C**, **G** Colony formation and EdU assays were performed to investigate the cell proliferation of vector and KIF18B overexpression cells with or without INK-128 treatment. Scale bar, 100 μm. **D**, **H** Colony formation and EdU assays were performed to investigate the cell proliferation of siNC and KIF18B silencing cells with or without MHY1485 treatment. Scale bar, 100 μm. **E** Cell migration of vector and KIF18B overexpression cells with or without INK-128 treatment were determined by Transwell assay. Scale bars, 100 μm. **F** Cell migration of siNC and KIF18B silencing cells with or without MHY1485 treatment were determined by Transwell assay. Scale bars, 100 μm. Error bars indicate the mean ± SD of three independent experiments. *P* values were calculated by student *t* test, *P < 0.05, **P < 0.01, ***P < 0.001.

As shown in Fig. 1, KIF18B expression was positively correlated with mTORC1 signaling in HCC tissues, suggesting that KIF18B might play a positive role in mTORC1 signal transduction. Silencing of KIF18B inhibited the activation of mTORC1 signaling and reduced the protein levels of mTOR, p70 S6K and 4EBP1 (Fig. 3A and S3A). Vice versa, KIF18B overexpression increased their protein and phosphorylation levels (Fig. 3A). Furthermore, KIF18B silencing accelerated the degradation of mTOR through ubiquitin–proteasome pathway (Fig. S3B, C). These results demonstrated that KIF18B regulated the activation of mTORC1 signaling and stability of mTOR, p70 S6K, and 4EBP1.

To investigate whether KIF18B promoted HCC progression through activating mTORC1 signaling, we treated KIF18B-depleted HCCLM3 cells with MHY1485, an agonist of mTORC1 signaling, and treated KIF18B overexpressing BEL-7402 cells with INK-128, an mTOR ATP site inhibitor. Western blot analyses on these cells showed that MHY1485 restored the phosphorylation of mTOR, p70 S6K and 4EBP1 in KIF18B-depleted HCCLM3 cells, while the KIF18B-induced phosphorylation of mTOR, p70 S6K and 4EBP1 were eliminated by INK-128 in BEL-7402 cells (Fig. 3B). Functional assays including EdU staining, colony formation and transwell assays showed that INK-128 treatment reduced the proliferative and migratory capacity enhanced by KIF18B overexpression in BEL-7402 cells (Fig. 3C, E, G). Consistently, MHY1485 treatment partially rescued the inhibitory effect of KIF18B silencing on proliferation and migration of HCC cells (Fig. 3D, F, H). Overall, these results suggest that KIF18B might function as an oncoprotein in HCC cells by enhancing mTORC1 signaling.

### KIF18B potentiates mTORC1 signaling via interacting with γ-Actin

**Fig. 4:**
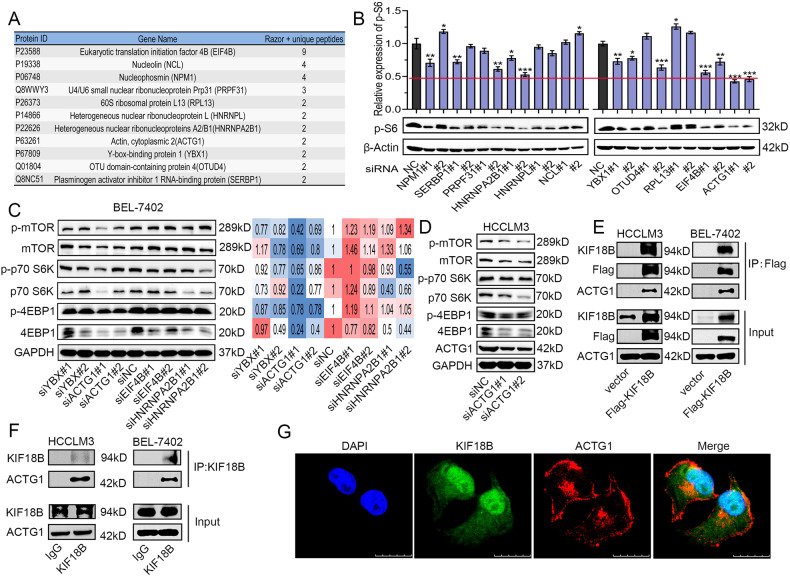
KIF18B potentiates mTORC1 signaling via interacting with γ-Actin. **A** The potential binding partners of KIF18B identified by mass spectrometry. **B** Western blot analysis of the phosphorylation of S6 after 11 genes knockdown in BEL-7402 cells. **C** Western blot analysis of the protein and phosphorylation levels of mTOR, p70 S6K, and 4EBP1 in YBX1, ACTG1, EIF4B, and HNRNPA2B1 silencing BEL-7402 cells. **D** Western blot analysis of the protein and phosphorylation levels of mTOR, p70 S6K, and 4EBP1 in ACTG1 silencing HCCLM3 cells. **E** Co-immunoprecipitation of γ-Actin with Flag-KIF18B. Flag-tagged proteins were immunoprecipitated using anti-Flag M2 agarose beads and immunoprecipitated proteins were detected with antibodies specific to KIF18B, ACTG1, and Flag. **F** Co-immunoprecipitation of endogenous KIF18B with γ-Actin. Immunoprecipitated proteins were detected with antibodies directed against KIF18B and ACTG1. **G** Representative immunofluorescence images showing co-localization of KIF18B and γ-Actin in HCC cells. Scale bar, 10 μm.

To elucidate the molecular mechanisms underlying the role of KIF18B in enhancing mTORC1 signaling, immunoprecipitation (IP) was performed to seek for potential interactors of KIF18B. The most abundant binding proteins identified by subsequent mass spectrometry was summarized in Fig. 4A. To pinpoint the proteins that modulate mTORC1 signaling from these binding partners, we silenced each of these 11 genes by 2 independent siRNAs (Fig. S4A), and the phosphorylation levels of S6 Ribosomal Protein at Ser235/236 (p S6), a marker of mTORC1 signaling activity were detected by western blot [26]. As shown in Fig. 4B, silencing of YBX1, ACTG1, EIF4B and HNRNPA2B1 reduced the level of p S6, among them, ACTG1 silencing resulted in the strongest decline. To further narrow down the candidates, the protein and phosphorylation levels of mTOR, p70 S6K and 4EBP1 were analyzed by western blot after silencing each of these 4 candidates in BEL-7402 cells. The result showed that only ACTG1 silencing could lead to the decline of the expression and phosphorylation levels of all 3 markers for mTORC1 signaling activity, which was similar to the phenotype caused by KIF18B silencing (Fig. 4C). Similar observations were obtained in HCCLM3 cells after silencing ACTG1 (Fig. 4D). These results suggest that ACTG1 might play a role in KIF18B-mediated augmentation of mTORC1 signaling.

ACTG1 encodes γ-Actin. Recent studies have reported that ACTG1 may be a biomarker for alcohol‑associated HCC, and it promotes HCC progression by regulating the cell cycle and cell apoptosis [27, 28]. To validate the interaction between KIF18B and γ-Actin, IP assays were performed. The results showed that both ectopic Flag-tagged KIF18B and endogenous KIF18B could interact with γ-Actin in HCCLM3 and BEL-7402 cells (Fig. 4E, F). IF staining of KIF18B and γ-Actin showed that these proteins co-localized in the cytoplasm, supporting our hypothesis that KIF18B could associate with γ-Actin in vivo (Fig. 4G). Notably, we observed that silencing of ACTG1 abolished the augmentation of proliferation and migration induced by the overexpression of KIF18B in BEL-7402 cells (Fig. S4B–E). Taken together, these results indicate that KIF18B regulates mTORC1 signaling via interacting with γ-Actin.

### γ-Actin and KIF18B regulate mTORC1 signaling through modulating lysosome positioning

**Fig. 5:**
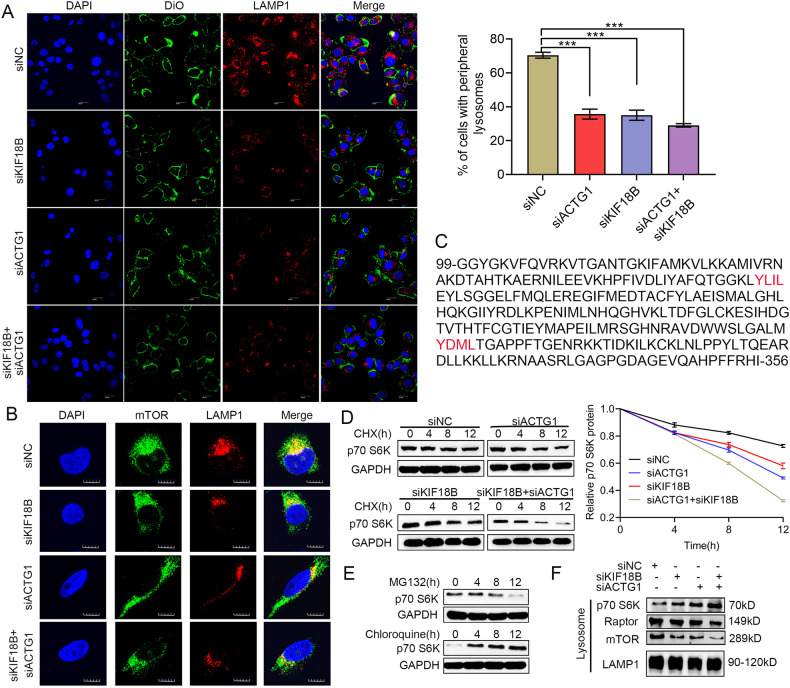
γ-Actin and KIF18B regulate mTORC1 signaling through modulating lysosomal positioning. **A** Immunofluorescence staining for LAMP1 (red) followed by counterstaining with DAPI (blue) and DiO (green) of NC, ACTG1 silencing, KIF18B silencing and simultaneous silencing cells. The merged images are also shown. Scale bar, 10 µm. Cells were categorized into perinuclear-dominant lysosomal distribution (>50% of LAMP1-positive signals localized in the perinuclear region, <5 µm from the nucleus) and peripheral-dominant distribution (>50% of LAMP1-positive signals localized in the peripheral region, >5 µm from the nucleus). Quantification is based on three independent experiments. **B** Immunofluorescence staining for mTOR (green) and LAMP1 (red) followed by counterstaining with DAPI (blue) of NC, ACTG1 silencing, KIF18B silencing, and simultaneous silencing cells. The merged images are also shown. Scale bar, 10 µm. **C** Analysis of p70 S6K amino acid sequence. p70 S6K contains 2 YXXθ motifs (labeled in red), which mediate sorting of proteins to lysosomes. **D** The half-life of p70 S6K in NC, ACTG1 silencing, KIF18B silencing and simultaneous silencing cells was detected by CHX chase assay. Relative p70 S6K protein level were graphed. **E** BEL-7402 cells were pretreated with CHX for 12 hours, and then the cells were treated with MG132 or chloroquine for the indicated times. Finally, expression of p70 S6K examined by western blot. **F** BEL-7402 cells were transfected with KIF18B siRNA, ACTG1 siRNA or both siRNA. 2 days post transfection, cells were subjected to western blot analysis of p70 S6K, mTOR, Raptor, and LAMP1 in lysosome fraction. Error bars indicate the mean ± SD of three independent experiments. P values were calculated by student *t* test, *P < 0.05, **P < 0.01, ***P < 0.001.

In mammal cells, the functional status of lysosomes is closely connected with cytoskeleton, since lysosomes are either localized around the microtubule organizing center (MTOC) (perinuclear lysosomes) or moving along microtubules (peripheral lysosomes) [29]. Lysosome movement towards the cell periphery is essential for cancer cell proliferation, invasion and metastasis [30, 31]. Meanwhile, mTORC1 must translocate to the lysosome surface and be activated by the small GTPase Rheb [32] and lysosomal positioning regulates mTORC1 signaling [33]. Since it has been reported that γ-Actin is involved in the reorientation of the MTOC [34] and kinesin can modulate the position and function of lysosome [35], we rationally proposed that KIF18B-γ-Actin interaction might influence mTORC1 translocation by modulating MTOC orientation and lysosome positioning. To test this hypothesis, we carried out IF staining to evaluate the distribution of lysosome after silencing KIF18B and ACTG1. The results demonstrated that the suppression of KIF18B and ACTG1 significantly reduced the percentages of cells with peripheral lysosomes and simultaneous silencing of both genes had synergistic effect (Fig. 5A). Consistently, the co-localization of mTOR and lysosome membrane marker LAMP1 was declined after silencing KIF18B and ACTG1 (Fig. 5B). Furthermore, we isolated lysosomes through density gradient centrifugation and performed western blot. As expected, KIF18B or ACTG1 silencing significantly reduced the protein levels of mTOR and raptor from lysosomes, and simultaneous silencing of both genes showed synergistic effect (Fig. 5F).

As shown in Figs. 3 and 4, KIF18B and ACTG1 also affected the stability of p70 S6K, and we found 2 YXXθ motifs in p70 S6K protein sequence (where Y represents tyrosine, X represents any amino acid and θ represents a bulky hydrophobic residue such as leucine), which mediate the sorting of both soluble and membrane-bound proteins to lysosomes [36], indicating that p70 S6K might be degraded through the lysosomal pathway (Fig. 5C). Therefore, we proposed that KIF18B-γ-Actin interaction might prevent p70 S6K from entering lysosomes for degradation via modulating lysosome positioning. CHX chase assay revealed that the silencing of KIF18B and ACTG1 synergistically accelerated the degradation of p70 S6K in BEL-7402 cells (Fig. 5D). Furthermore, the treatment of lysosomal inhibitor (chloroquine) significantly increased the protein level of p70 S6K, while proteasomal inhibitor (MG132) treatment did not, which confirmed that p70 S6K was mainly degraded through the lysosomal pathway (Fig. 5E). Meanwhile, the protein level of p70 S6K in lysosomes was significantly higher in KIF18B-depleted and ACTG1-depleted cells, and simultaneous silencing of both genes showed synergistic effect (Fig. 5F). Overall, these data demonstrated that γ-Actin and KIF18B could cooperatively regulate mTORC1 signaling through modulating lysosome positioning.

### FOXM1 promotes KIF18B expression in HCC

**Fig. 6:**
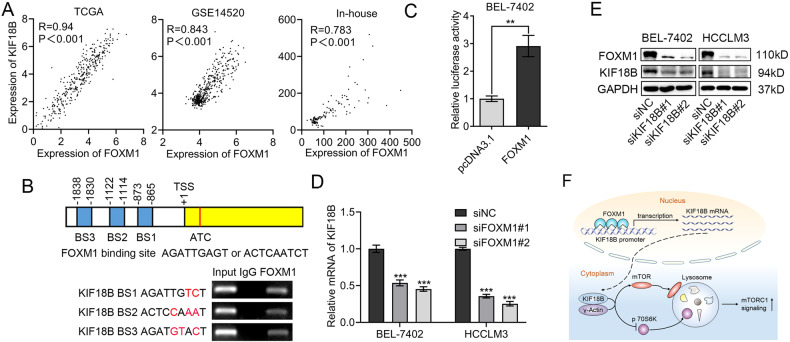
FOXM1 promotes KIF18B expression in HCC. **A** Pearson correlation analysis of KIF18B and FOXM1 expressions in in-house, TCGA, and GSE14520 datasets. **B** Schematic illustration of three potential FOXM1-binding sites (BS1, BS2, and BS3) in the KIF18B promoter (top). The 9-base pair sequence of the FOXM1 consensus site and sequences of three FOXM1-binding sites in the KIF18B promoter are shown. Chromatin was isolated from BEL-7402 cells, and ChIP assay was performed with rabbit IgG control and FOXM1-specific antibody (bottom). **C** Full-length KIF18B luciferase construct was transfected into BEL-7402 cells with empty vector or the FOXM1 overexpression plasmid for 48 h. Transcriptional activation was analyzed with the dual luciferase reporter assay. **D**, **E** qRT-PCR (**D**) and western blot (**E**) analysis of KIF18B expression in FOXM1 silencing HCC cells. **F** The proposed model of the upregulation of KIF18B in HCC and the function of KIF18B in the activation of mTORC1 signaling. Error bars indicate the mean ± SD of three independent experiments. *P* values were calculated by student *t* test, *P < 0.05, **P < 0.01, ***P < 0.001.

To elucidate the mechanism for the upregulation of KIF18B expression in HCC, we focused on transcription factors that might activate KIF18B expression. Analysis of the correlation between the expression of reported transcription factors and KIF18B expression in TCGA dataset revealed that the expression of FOXM1, a transcription factor reported to be overexpressed in HCC was mostly correlated with KIF18B expression, with the highest *R* value of 0.94 (Fig. 6A). Moreover, the expression of FOXM1 was also positively correlated with KIF18B expression in the in-house and GSE14520 datasets (Fig. 6A). To confirm KIF18B as a target gene of FOXM1, we analyzed KIF18B promoter by MatInspector program (Genomatix) and found 3 potential binding sites of FOXM1, which we validated by ChIP assay (Fig. 6B). Then, we constructed a luciferase reporter vector containing the promoter region of KIF18B to evaluate the regulatory effect of FOXM1 on KIF18B expression. In BEL-7402 cells, FOXM1 overexpression increased luciferase activity by threefold (Fig. 6C). Consistently, siRNA-mediated FOXM1 silencing resulted in substantial reduction in both mRNA and protein levels of KIF18B in BEL-7402 and HCCLM3 cells (Fig. 6D, E). Collectively, these data suggest that KIF18B is a direct transcriptional target of FOXM1 in HCC.

## Discussion

In this study, we found that the expression of KIF18B was positively correlated with mTORC1 signaling in HCC tissues, suggesting that KIF18B might play a role in HCC recurrence through activating mTORC1 signaling. Our in vitro and in vivo functional assays demonstrated that KIF18B promoted proliferation and migration, while it inhibited apoptosis of HCC cells. Further investigation revealed that KIF18B interacted with γ-Actin and they cooperatively stimulated lysosome movement towards the cell periphery, promoted mTORC1 translocation to lysosome membrane, and prohibited p70 S6K from entering lysosomes, which eventually led to the activation of mTORC1 signaling. Finally, we discovered that KIF18B was a direct target of FOXM1, whose overexpression led to enhanced transcriptional activity of KIF18B in HCC.

Kinesin superfamily proteins (KIFs) are classified into 14 families based on structural differences and 45 KIFs have been discovered in humans [37]. Many KIFs have been implicated in HCC [38, 39] and they regulate the progression of HCC in different ways. For instance, KIF15 interacted with phosphoglycerate dehydrogenase (PHGDH) and stabilized it, thus promoting cancer stem cell phenotype and malignancy via PHGDH-mediated reactive oxygen species imbalance in HCC [40]. The functional studies of KIF18B have been focused on spindle assembly and chromosome alignment, and KIF18B plays pivotal roles during mitosis [18]. Meanwhile, it has been implicated in many cancers such as cervical cancer, PDAC and colon adenocarcinoma, where its expression tends to be remarkably upregulated compared with matched normal tissues [21, 23, 25]. In a study involving human prostate cancer cells, KIF18B was shown to promote prostate cancer progression by activating mTOR signaling pathway [41]. Even though this study did not elucidate the molecular mechanism, the similarity between this study and our study regarding KIF18B’s involvement in mTOR signaling reinforces the credibility of our research and indicates that such regulation of mTOR signaling by KIF18B might be universal in various types of cancer.

Recently, 2 groups have reported the upregulation of KIF18B and its oncogenic potential in HCC [22, 42]. In Qiu et al.’s study, RNA-based bioinformatics analysis identified KIF18B as a regulator in HCC microenvironment, but the underlying molecular mechanism by which KIF18B modulates HCC microenvironment required further experimental investigation [42]. Yang et al.’s study suggested that KIF18B might contribute to HCC progression through activating Wnt/β-catenin pathway, however the detailed molecular mechanism remained unclear [22]. Interestingly, our study might shed some light on the mechanisms underlying their observations. It has been demonstrated that mTORC1 signaling is critical for the transformation of tumor microenvironment by promoting angiogenesis and reprogramming metabolic pathway [43]. Therefore, it is valuable to investigate whether KIF18B modulates HCC microenvironment through enhancing mTORC1 signaling activity. Meanwhile, β-catenin, an important component of Wnt/β-catenin pathway, was reported to degrade through the lysosomal pathway [44], and our work suggests that KIF18B might enhance the stability of β-catenin by modulating lysosome positioning, and finally activate the Wnt/β-catenin pathway.

A few other members of kinesin family have been implicated in malignancy and drug resistance of tumor, indicating kinesin proteins could be promising targets for tumor treatment [45]. For instance, Filanesib, an inhibitor of KIF11, recently completed Phase 2 clinical trial for multiple myeloma treatment, with a response rate of 16% and progression free survival value of 1.6 months [46], now Filanesib has been scheduled to enter Phase 3 clinical trials for multiple myeloma [47]. As shown by our study as well as researches of other groups, KIF18B functions as an oncogenic protein in various types of cancer, thus developing KIF18B inhibitors would also be a promising anticancer strategy. Moreover, upregulated mTORC1 signaling activity plays a pivotal role in HCC, and single mTOR inhibitor or the combination of mTOR inhibitors with other drugs have been used in clinical trials for the treatment of HCC [48]. mTOR inhibitors have been proved to decrease HCC recurrence rate after liver transplantation [49, 50]. Our research suggests that the combination of mTOR inhibitors and KIF18B inhibitors may synergistically enhance their preventing recurrence and anti-tumor effect.

In summary, the results of this study demonstrate that KIF18B is a direct target of FOXM1 and promotes HCC recurrence through activating mTORC1 signaling. Mechanistically, KIF18B interacts with γ-Actin. They synergistically modulate lysosome positioning, subsequently promote mTORC1 translocation to lysosome membrane, and prevent p70 S6K from entering lysosomes for degradation (Fig. 6F). Our study highlights the potential of KIF18B as a therapeutic target for the treatment of HCC.

## Materials and methods

### Cell lines and clinical samples

Hep3B, Huh1, Huh7, LO2 and HEK293T were obtained from American Type Culture Collection. HCCLM3, BEL-7402 and SMMC-7721 were purchased from Cell Bank of Chinese Academy of Sciences. The cell lines HCCLM3, BEL-7402, Hep3B, Huh1, Huh7 and 293 T were cultured in Dulbecco’s Modified Eagle Medium (DMEM) supplemented with 10% fetal bovine serum (FBS) (Biological Industries, 04-001-1), 100 U/ml penicillin and 0.1 mg/ml streptomycin. LO2 and SMMC-7721 cell lines were maintained in Roswell Park Memorial Institute-1640 (RPMI-1640) medium supplemented with 10% FBS, 100 U/ml penicillin and 0.1 mg/ml streptomycin. All of these cells were incubated at 37 °C in an atmosphere of 5% CO_2_. A total of 60 HCC tissues were obtained from the affiliated hospital of Hangzhou Normal University. Written informed consent was obtained from each patient, and the study was approved by the Biomedical Ethics Committee of the affiliated hospital of Hangzhou Normal University. This study was performed in accordance with the Declaration of Helsinki.

### Lentivirus infection and in vivo models

HCCLM3 or BEL-7402 cells were infected with lentiviruses in presence of polybrene and selected with 2 μg/mL and 1 μg/mL puromycin, respectively, for 7 days. For the subcutaneous xenograft model, five-week-old male BALB/c nude mice were used. All animal care and handling procedures were performed in accordance with the National Institutes of Health guide for the care and use of Laboratory animals and approved by the Animal Care and Use Committee of Hangzhou Normal University. The control and experimental HCCLM3 cells (5 × 10^6^) were suspended in 100 μL PBS and then injected subcutaneously into the flanks of the nude mice. Tumor growth was examined over the course of 4 weeks and tumor volume was measured with calipers and calculated using the formula: Volume (mm^3^) = length × width^2^/2 as previously reported [51]. After 4 weeks, the mice were sacrificed and the tumors were weighed, fixed, and paraffin-embedded for further analysis.

### Statistical analysis

Spearman test was used to analyze the correlation between KIF18B and p-mTOR expression in HCC tissues. The Kaplan-Meier method was used to analyze patient survival. The Student, *t* test was used to for the comparisons between two groups. Statistical analysis and graphs were generated using the SPSS 20.0 software and GraphPad Prism 7 software. *P* < 0.05 was considered as significant.

### Supplementary information

supplemental material

supplemental figure 1

supplemental figure 2

supplemental figure 3

supplemental figure 4

Original Data File

## Data Availability

The authors declare that all data in the article are available.
