# Supplementary material for: Kinesin family member 18B activates mTORC1 signaling via actin gamma 1 to promote the recurrence of human hepatocellular carcinoma
Source: Oncogenesis. 2023 Nov 13;12(1):54. doi: 10.1038/s41389-023-00499-7 (PMC10643429; doi:10.1038/s41389-023-00499-7)
Supplement: Supplementary file 1 — supplemental material [file 41389_2023_499_MOESM1_ESM.docx]

**Supplemental files for**

**Kinesin family member 18B** **activates mTORC1 signaling via Actin gamma 1 to promote the recurrence of human hepatocellular carcinoma**

Qian Li, Mengqing Sun, Yao Meng, Mengqing Feng, Menglan Wang, Cunjie Chang, Heng Dong, Fangtian Bu, Chao Xu, Jing Liu, Qi Ling, Yiting Qiao, Jianxiang Chen

**Supplemental materials and methods**

**siRNA interference and transfection**

For siRNA interference, siRNAs were transfected into HCC cells using Lipofectamine RNAiMAX (Invitrogen, 13778150). The siRNAs were purchased from GenePharma (Shanghai, China) and the sequences of siRNAs are listed in supplementary table 1.

**Immunohistochemistry (IHC)**

The sections were deparaffinized and rehydrated, and endogenous peroxidase was inactivated with 0.3% H_2_O_2_/methanol. For antigen retrieval, slides were boiled in 0.01 M sodium citrate buffer (pH 6.0) for 30 min. After blockage with 5% bovine serum albumin (BSA), slides were incubated with primary antibodies diluted in blocking buffer at 4 ℃ overnight, followed by washing and the incubation with secondary antibody. After thorough washing to remove unbound antibodies, signals were developed with DAB Immunohistochemistry Color Development Kit (Sango Biotech, E670033). The sections were viewed and scored separately by two pathologists blinded to the clinical parameters. The primary antibodies and their dilution ratios are listed as follows: KIF18B (1:800, Bethyl Laboratories, A303-982A); Ki-67(1:200, Sigma Aldrich, SAB5300423); p-mTOR (1:100, Cell Signaling Technology, 2976S).

**Western blot and immunoprecipitation (IP)**

Total cellular proteins were extracted using RIPA lysis buffer containing protease inhibitor (Roche, 04693132001) and phosphatase inhibitor (Roche, 04906837001). Equal amounts of total protein from various samples were separated with SDS–PAGE and transferred to PVDF membranes, which were blocked with 5% skimmed milk in PBST for 1 h, and incubated with primary antibodies overnight at 4 °C. The membranes were then washed and incubated with secondary antibodies for 1 h at room temperature. Protein visualization was performed using Western Bright Sirius kit (Advansta, K-12043). The primary antibodies and their dilution ratios are listed as follows: KIF18B(1:1000, Bethyl Laboratories, A303-982A); GAPDH (1:5000, Cell Signaling Technology, 2118S); Flag (1:5000, Sigma Aldrich, F3165); cleaved-PARP (1:500, Santa Cruz, SC56196); cleaved-caspase 3 (1:500, Cell Signaling Technology, 9664S); mTOR (1:1000, Cell Signaling Technology, 2972S); p-mTOR (1:1000, Cell Signaling Technology, 5536S); p70 S6K (1:1000, Cell Signaling Technology, 9202S); p-p70 S6K (1:1000, Cell Signaling Technology, 9205S); 4EBP1 (1:1000, Cell Signaling Technology, 9644S); p-4EBP1 (1:1000, Cell Signaling Technology, 2855S); ACTG1 (1:500, Santa Cruz, SC8432); LAMP1 (1:1000, Cell Signaling Technology, 9091P) ; p-S6(1:1000, Cell Signaling Technology, 4857S); β-actin(1:5000, Abcam, ab8226) and FOXM1(1:2000, Thermo Fisher , PA5-27144); Raptor(1:500, Proteintech, 20984-1-AP). For the IP, primary antibody (2μg) was added into 1000 μg of total protein, and incubated overnight on a rotary shaker at 4 °C. The protein A/G beads (Invitrogen, 10002D/10004D) were added to the mixture and rotated for 1 h at 4 °C. The precipitates were then washed and boiled in order to retrieve the bound proteins. The samples were then analyzed with Western blot and mass spectrometry (MS). MS was performed by LC Science, in Hangzhou, China.

**Luciferase reporter assay and chromatin immunoprecipitation (ChIP)**

The promoter sequence of KIF18B was amplified by PCR and inserted into the pGL3 basic vector. The coding sequence of FOXM1c was amplified by PCR and then inserted into the pcDNA3.1 vector. pGL3 basic reporter plasmid and the internal control plasmid pRL-TK were transfected into BEL-7402 cells grown to 70% confluence. The FOXM1 expression plasmid or empty vector were co-transfected for 48 h, and reporter activity was assayed using the Dual-Glo Luciferase Assay System (Promega, E2920). For ChIP assay, BEL-7402 cells grown to 90% confluence were cross-linked with 1% (v/v) formaldehyde. Chromatin was sonicated into fragments of 200 to 800 bp over 30 cycles of 30s on /30s off using a Bioruptor Sonicator (Diagenode, Bioruptor Plus). The lysates were pre-cleared with protein A/G beads and incubated with anti-FOXM1 antibody (Thermo Scientific, PA5-27144) or control IgG overnight at 4 °C. Protein A/G beads were added to the mixture and rotated at 4 °C for 1 h. After washing, the DNA was eluted, and reverse cross-linked overnight at 65 °C. Eluted DNA was purified using Gel & PCR Clean Up Kit (OMEGA, D2500) and used as templates for RT-PCR. The primers used to amplify KIF18B promoter sequences are listed in supplementary table 2.

**Lysosome purification**

Lysosomes were isolated according to the protocol of Lysosome Enrichment Kit for Tissues and Cultured Cells (Invitrogen, 89839). Briefly, 50-200 mg cell pellet was lysed in 800μL Reagent A and sonicated on ice. Add 800μL Reagent B, mix and centrifuge to collect supernatant. In an ultracentrifuge tube, prepare a discontinuous density gradient. Mix the supernatant with the Cell Separation Media to make a final concentration of 15% Media. Overlay the sample containing the 15% Media on top of the density gradient, ultracentrifuge the sample at 145,000 × g for 2 hours at 4°C. The lysosome band is located in the top 2mL of the gradient.

**Cell Counting Kit-8 (CCK8), colony formation and EdU-DNA synthesis assays**

Briefly, HCC cells were seeded at a density of 1 × 10^3^ cells/well in 96-well plates. CCK 8 solution (Meilunbio, MA0218) was added to each well at a final concentration of 10% and incubated for 2 h. Then the absorbance of the samples was measured at 450 nm using a Multiskan FC microplate reader every 24 h for 5 days. For the colony formation assay, 5 × 10^3^ cells/ well were seeded in 6-well plates. After 12 days, the visible colonies were fixed with 4% formaldehyde and stained with crystal violet. The colonies were imaged with a scanner and quantified using ImageJ software. For the EdU-DNA synthesis assay, HCC cells were seeded in 96-well plates at a density of 3 × 10^4^ per well. After 24 h, the culture medium was replaced with culture medium containing 50 μM EdU and kept for 2 h. The cells were then processed with the Cell-light EdU Apollo 567/488 in vitro Kit (Ribobio, C10310) according to the manufacturer’s instructions. Images were acquired with an inverted fluorescence microscope (Nikon, TS2-FL) and analyzed with ImageJ software.

**Migration assay**

Cell migration assay was performed using transwell plates (Corning, 3422). Briefly, 2 × 10^5^ cells/well were re-suspended with serum-free medium in the top chamber of the transwell plates, and medium with 10% FBS was added to the bottom chamber. After 24 h, the cells that had migrated across the membrane were fixed with 4% paraformaldehyde, stained with crystal violet. Migrated cells were counted in three randomly selected fields under microscope.

**Immunofluorescence (IF)**

The cells were seeded on glass coverslips, fixed with 4% paraformaldehyde for 10 min, and permeabilized in 0.1% Triton X-100 for 10 min at room temperature. The cells were then blocked with 5% BSA in PBS for 30 min at room temperature and incubated with primary antibodies overnight at 4 °C. Then, the cells were washed and incubated with Alexa Fluor 488 goat anti-rabbit IgG or Alexa Fluor 594 goat anti-mouse IgG secondary antibodies (Invitrogen, A11008 and A11005) for 1 h at room temperature. The nuclei were counterstained with DAPI for 10 min. Fluorescent images were captured using a Confocal Microscope (Olympus, FV3000). The primary antibodies and their dilution ratios are listed as follows: KIF18B (1:500, Bethyl Laboratories, A303-982A); ACTG1 (1:500, Abcam, ab123034); LAMP1 (1:100, Cell Signaling Technology, 15665T); mTOR (1:200, Cell Signaling Technology, 2983T) and mTOR (1:500, Proteintech, 66888-1).

**Quantitative RT-PCR (qRT-PCR)**

Total RNA was extracted with Trizol (Invitrogen, 15596026) and cDNA was synthesized using the HiScript III 1st Strand cDNA Synthesis Kit (Vazyme, R323) according to the manufacturer’s instructions. ChamQ Universal SYBR qPCR Master Mix (Vazyme, Q711) was used to conduct qRT-PCR. Primer sequences are listed in supplementary table 3.

**Cycloheximide (CHX) chase assay**

Cells were incubated with CHX to inhibit protein synthesis for 0h, 4h, 8h and 12h respectively. Then cells were harvested, lysed with RIPA lysis buffer. Equal amounts of protein were loaded for Western blot analysis of p70 S6K. The intensity of protein bands was quantitated by ImageJ software.

**Supplemental Tables**

Table S1. Sequences of siRNAs

| Gene | Sequence |
| --- | --- |
| siKIF18B#1 | CAGCUACCAGGAGGUGUAUAA |
| siKIF18B#2 | AUGCCAUCUUCCAGAUCUUUG |
| siEIF4B#1 | GACCGCUAUGAAGACCGAUAU |
| siEIF4B#2 | GACAAGUAUCGAGAUCGUUAU |
| siNPM1#1 | GCGCCAGUGAAGAAAUCUAUA |
| siNPM1#2 | CCUAGUUCUGUAGAAGACAUU |
| siRPL13#1 | CCGCAGAACAGGAUGUUGAAA |
| siRPL13#2 | GAGGAAGAGAAGAAUUUCAAA |
| siPRPF31#1 | CAAGUGCAAGAACAAUGAGAA |
| siPRPF31#2 | GCACCCAACCUGUCCAUCAUU |
| siFOXM1#1 | GCACUAUCAACAAUAGCCUAU |
| siFOXM1#2 | GGAAAUGCUUGUGAUUCAACA |
| siACTG1#1 | CCGAGCCGUGUUUCCUUCCAU |
| siACTG1#2 | CGCAUCCUCCUCUUCUCUGGA |
| siHNRNPL#1 | GCCGACAACCAAAUAUACAUU |
| siHNRNPL#2 | CCUCAACAACAACUUCAUGUU |
| siHNRNPA2B1#1 | CAGAAAUACCAUACCAUCAAU |
| siHNRNPA2B1#2 | GCUUCUUCCUAUUUGCCAUGG |
| siYBX1#1 | AGCAGACCGUAACCAUUAUAG |
| siYBX1#2 | CCAGUUCAAGGCAGUAAAUAU |
| siOTUD4#1 | GAUAUUGUGUAUCCCAUAAAG |
| siOTUD4#2 | GCGUUUAUAGAAGGAUCAUUU |
| siSERBP1#1 | CCUGAAGGUGAAGAACAUCAU |
| siSERBP1#2 | GCCGAGGAGAUGGAUUUGAUU |
| siNCL#1 | AGUAAAGGGAUUGCUUAUAUU |
| siNCL#2 | CGGUGAAAUUGAUGGAAAUAA |
| siNC | UUCUCCGAACGUGUCACGU |

Table S2. Sequences of RT-PCR primers

| KIF18B-CHIP-BS1-F | CCCTACCAAGACCTCTCAGC |
| --- | --- |
| KIF18B-CHIP-BS1-R | TAGGGAGATTGGGAGCTTGC |
| KIF18B-CHIP-BS2-F | CTTGGGGAGCCAAAACCTCT |
| KIF18B-CHIP-BS2-R | TGGAGGTGCTGGTCATTGTA |
| KIF18B-CHIP-BS3-F | GGCAAGTCTGATGAGGGAGT |
| KIF18B-CHIP-BS3-R | TTCCAACGTCCCCAGCATAT |

Table S3. Sequences of qRT-PCR primers

| KIF18B Forward | AGAAGGGCAAAGACCTGACG |
| --- | --- |
| KIF18B Reverse | ACACTGAGCAGTTGTAGCCC |
| EIF4B Forward | GGCAAAGCCTGTTGACACAGCT |
| EIF4B Reverse | TTCACTTCGCCAGCTTGGGTGT |
| NPM1 Forward | GCCAGTGCATATTAGTGGACAGC |
| NPM1 Reverse | GGAACCTTGCTACCACCTCCAG |
| RPL13 Forward | CGCTCCAAACTCATCCTCTTCC |
| RPL13 Reverse | CTCTTCCTCAGTGATGACTCGAG |
| PRPF31 Forward | GCTTCTCGTCTACCTCAGTGCT |
| PRPF31 Reverse | TGTGCTCTCGTGGAAACTGTCC |
| ACTG1 Forward | ATGGAAGAAGAGATCGCCGC |
| ACTG1 Reverse | CCCGACGATGGAAGGAAACA |
| HNRNPL Forward | GTGTGGTGGAAGCAGACCTTGT |
| HNRNPL Reverse | CAAACTCCACCAGTGCTTGTCTC |
| HNRNPA2B1 Forward | GGAGGCAACTTTGGCTTTGG |
| HNRNPA2B1 Reverse | CATCCCCAAATCCACGTCCA |
| YBX1 Forward | GCAGGAGAACAAGGTAGACCAG |
| YBX1 Reverse | CTTCATTGCCGTCCTCTCTAGG |
| OTUD4 Forward | TCCACAGGAATGGGTAGGAC |
| OTUD4 Reverse | AGAGACTGACACATAGCAGAGC |
| SERBP1 Forward | AGAAAGGCGACCACCTCGTGAA |
| SERBP1 Reverse | ACCTCTTCCAAGACCACCACGA |
| NCL Forward | GCCTGTCAAAGAAGCACCTGGA |
| NCL Reverse | GAAAGCCGTAGTCGGTTCTGTG |

**Supplemental Figures**

**Fig. S1. KIF18B inhibits HCC cell apoptosis.** A, Gene Set Enrichment Analysis for microarray datasets of HCC samples in GSE14520. B, qRT-PCR analysis of KIF18B mRNA expression in normal human hepatic cell line and HCC cell lines. C and D, Flow cytometric analysis of apoptosis in KIF18B silencing (C) and overexpression (D) HCC cells. Error bars indicate the mean ± SD of three independent experiments. P values were calculated by student t-test, ^*^P<0.05, ^**^P<0.01, ^***^P<0.001. E, Expression of KIF18B, cleaved caspase 3 and cleaved PARP in KIF18B silencing and ectopic expression HCC cells examined by Western blot.

**Fig. S2. KIF18B and mTOR co-expressed in HCC tissues.** A, Immunofluorescence detection of KIF18B and mTOR in HCC tissues. Scale bars, 10μm.

**Fig. S3. KIF18B activates mTORC1 signaling and stabilizes mTOR.** A, Western blot analysis of the phosphorylation and total protein levels of mTOR, p70 S6K and 4EBP1 in the KIF18B-depleted HCC cells. B, Western blot analysis of mTOR after DMSO, C381(5µM) and PR-619(15µM) treatment. C, The NC and KIF18B silencing cells were treated with PR-619 at indicated time, and the expression of mTOR was detected by western blot.

**Fig. S4****.** **KIF18B promotes cancer properties via ACTG1 in HCC.** A, Validation of the knockdown efficiencies of 11 genes. B, Western blot analysis of KIF18B and ACTG1 expression after transfection. C and D, Colony formation(C) and EdU (D) assays were performed to investigate the cell proliferation of vector and KIF18B overexpression cells with or without ACTG1 silencing. E, Cell migration of vector and KIF18B overexpression cells with or without ACTG1 silencing was determined by Transwell assay. Error bars indicate the mean ± SD of three independent experiments. P values were calculated by student t-test, ^*^P<0.05, ^**^P<0.01, ^***^P<0.001.
