# Supplementary figures and images for: Kinesin family member 18B activates mTORC1 signaling via actin gamma 1 to promote the recurrence of human hepatocellular carcinoma
Source: Oncogenesis. 2023 Nov 13;12(1):54. doi: 10.1038/s41389-023-00499-7 (PMC10643429; doi:10.1038/s41389-023-00499-7)

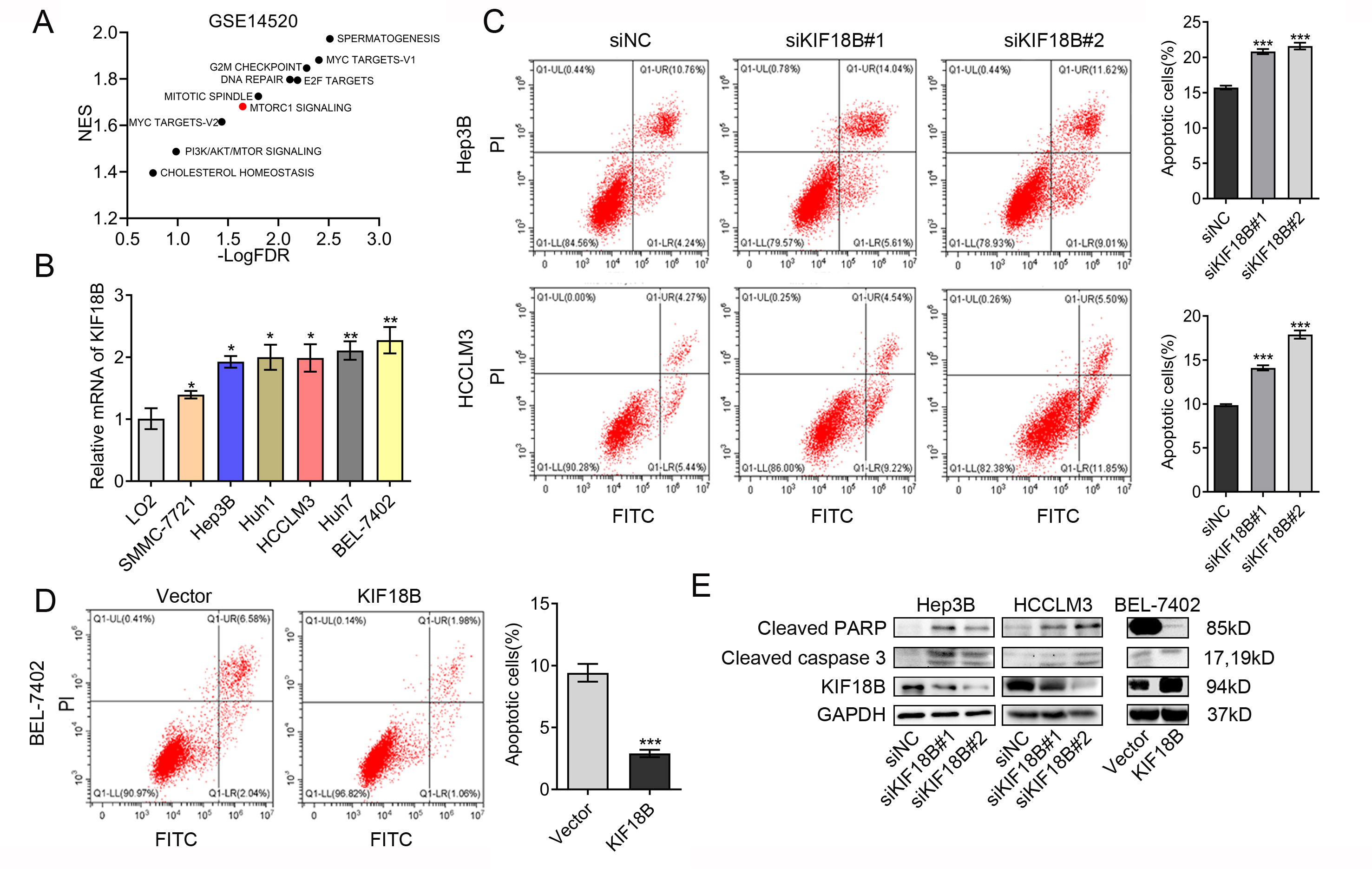

Supplement: Supplementary file 2 — supplemental figure 1 [file 41389_2023_499_MOESM2_ESM.jpg]

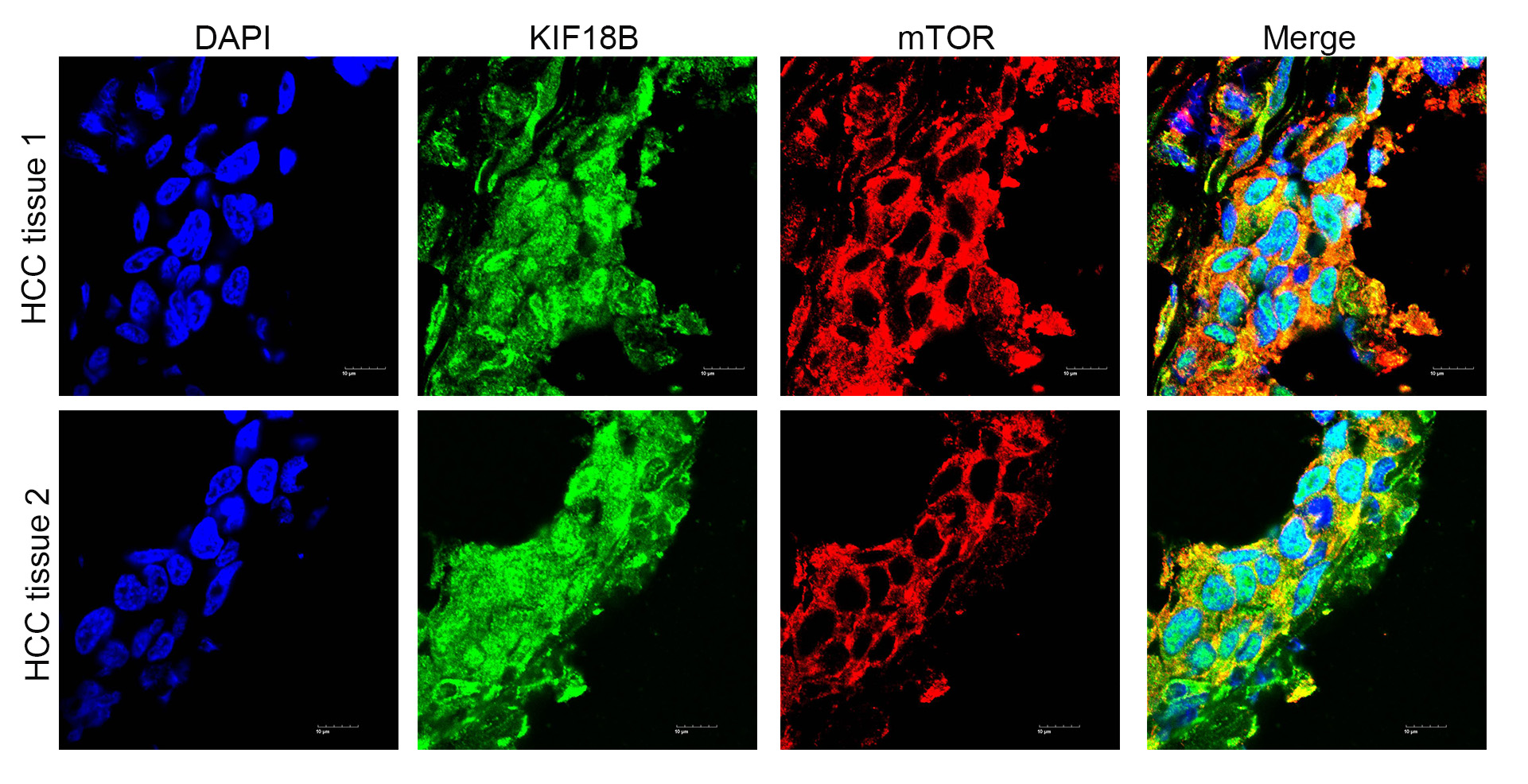

Supplement: Supplementary file 3 — supplemental figure 2 [file 41389_2023_499_MOESM3_ESM.jpg]

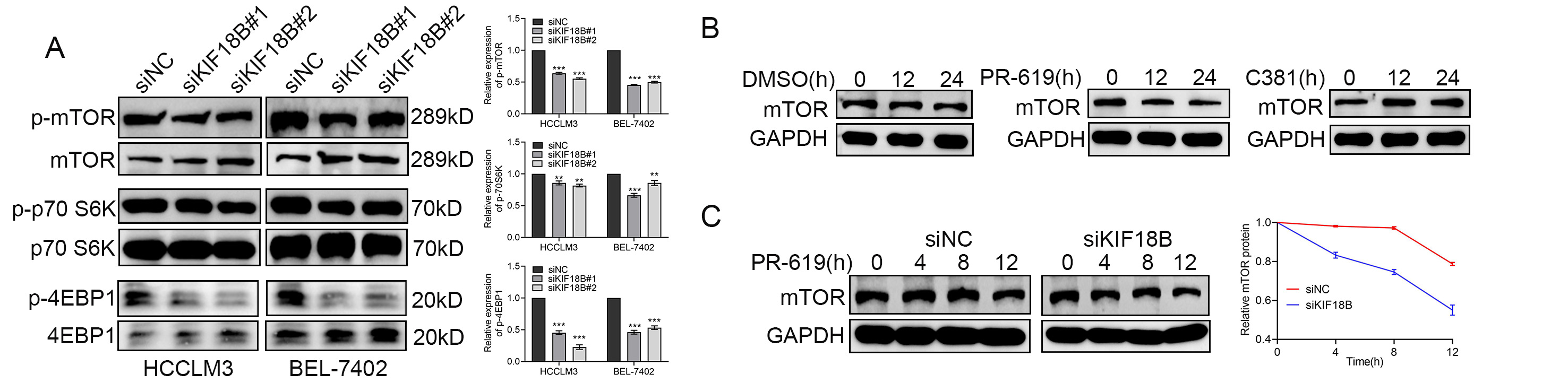

Supplement: Supplementary file 4 — supplemental figure 3 [file 41389_2023_499_MOESM4_ESM.jpg]

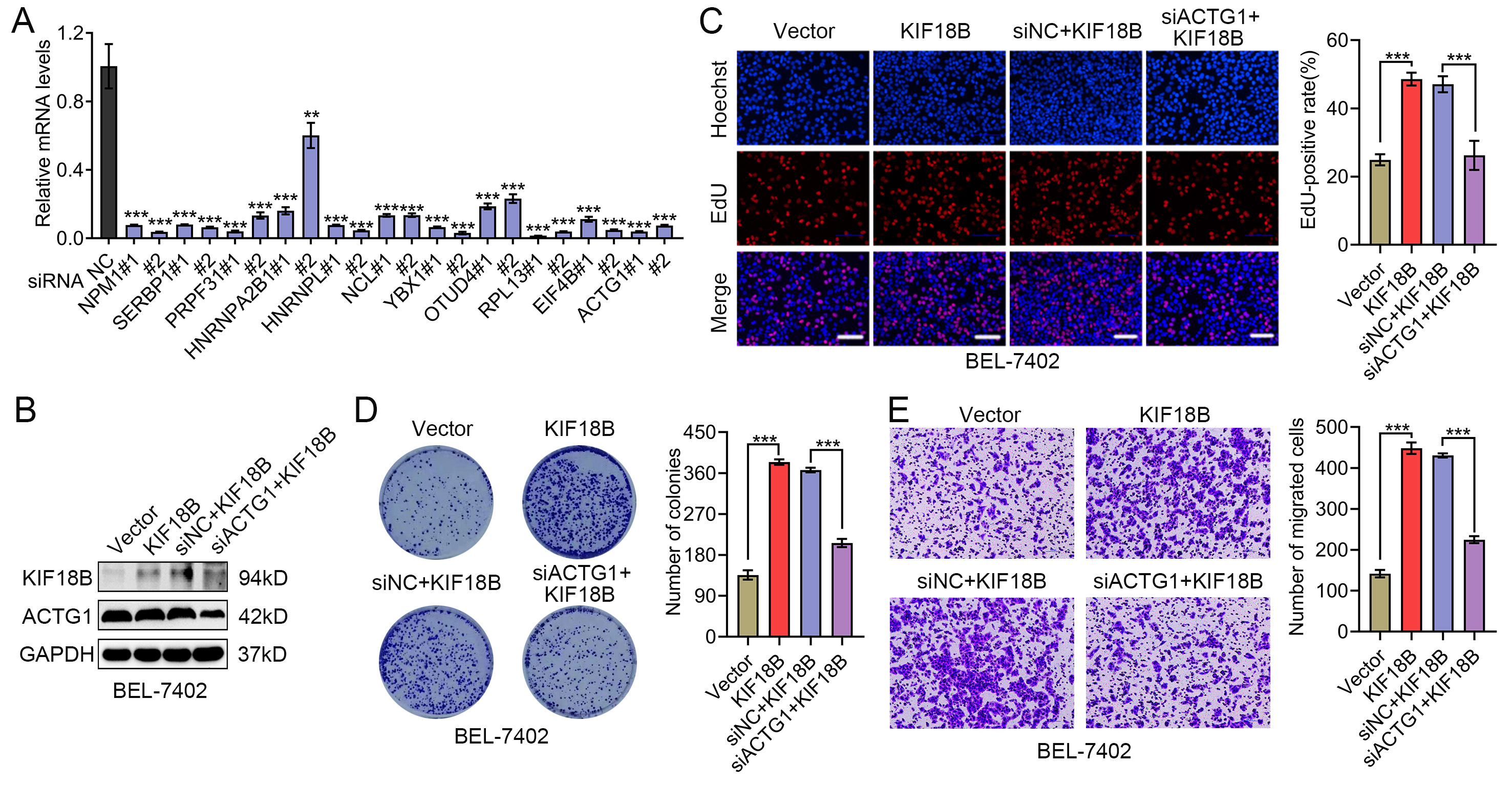

Supplement: Supplementary file 5 — supplemental figure 4 [file 41389_2023_499_MOESM5_ESM.jpg]

## Slide 1
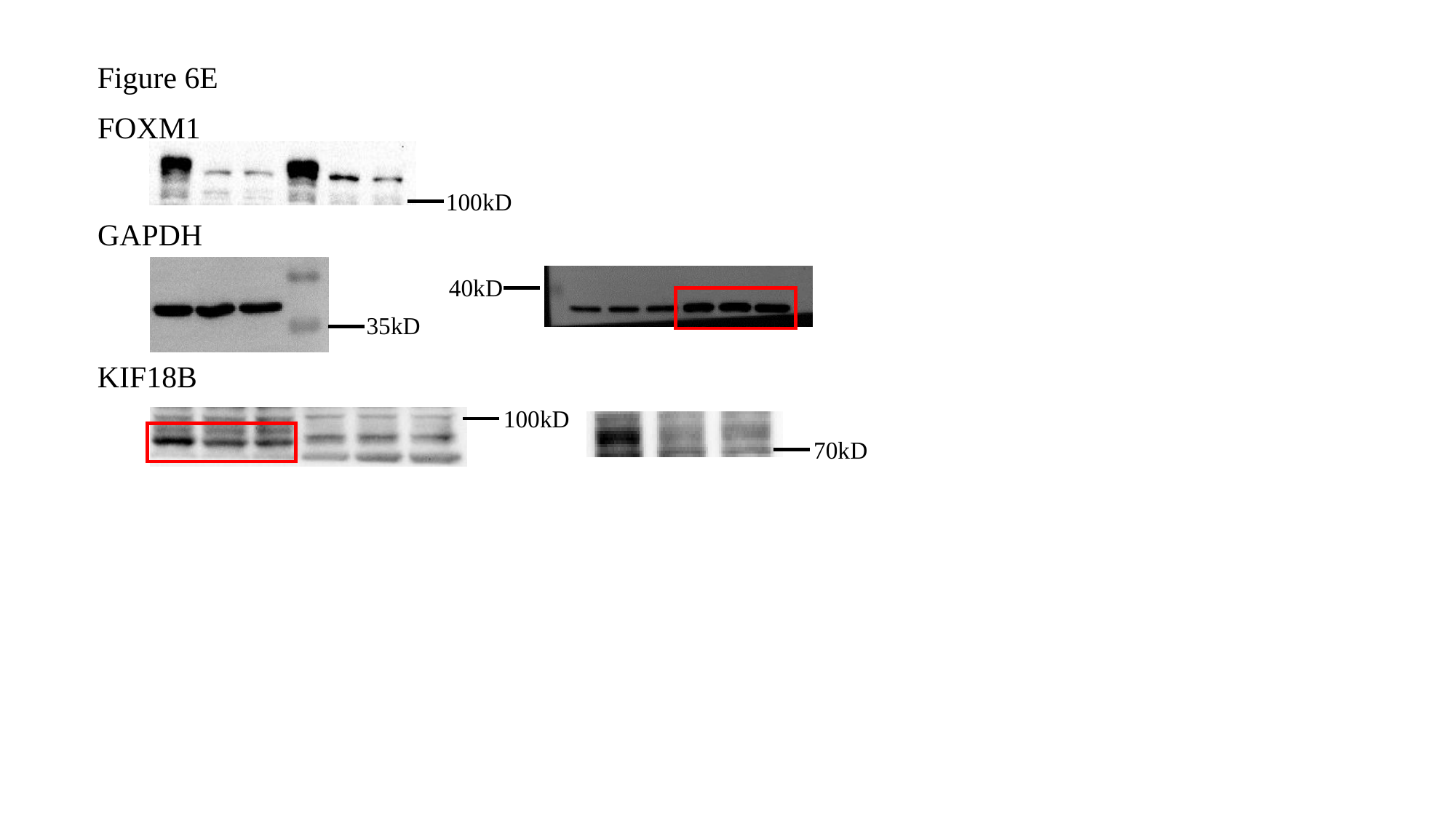

Figure 6E
FOXM1
100kD
GAPDH
40kD
35kD
KIF18B
100kD
 70kD

## Slide 2
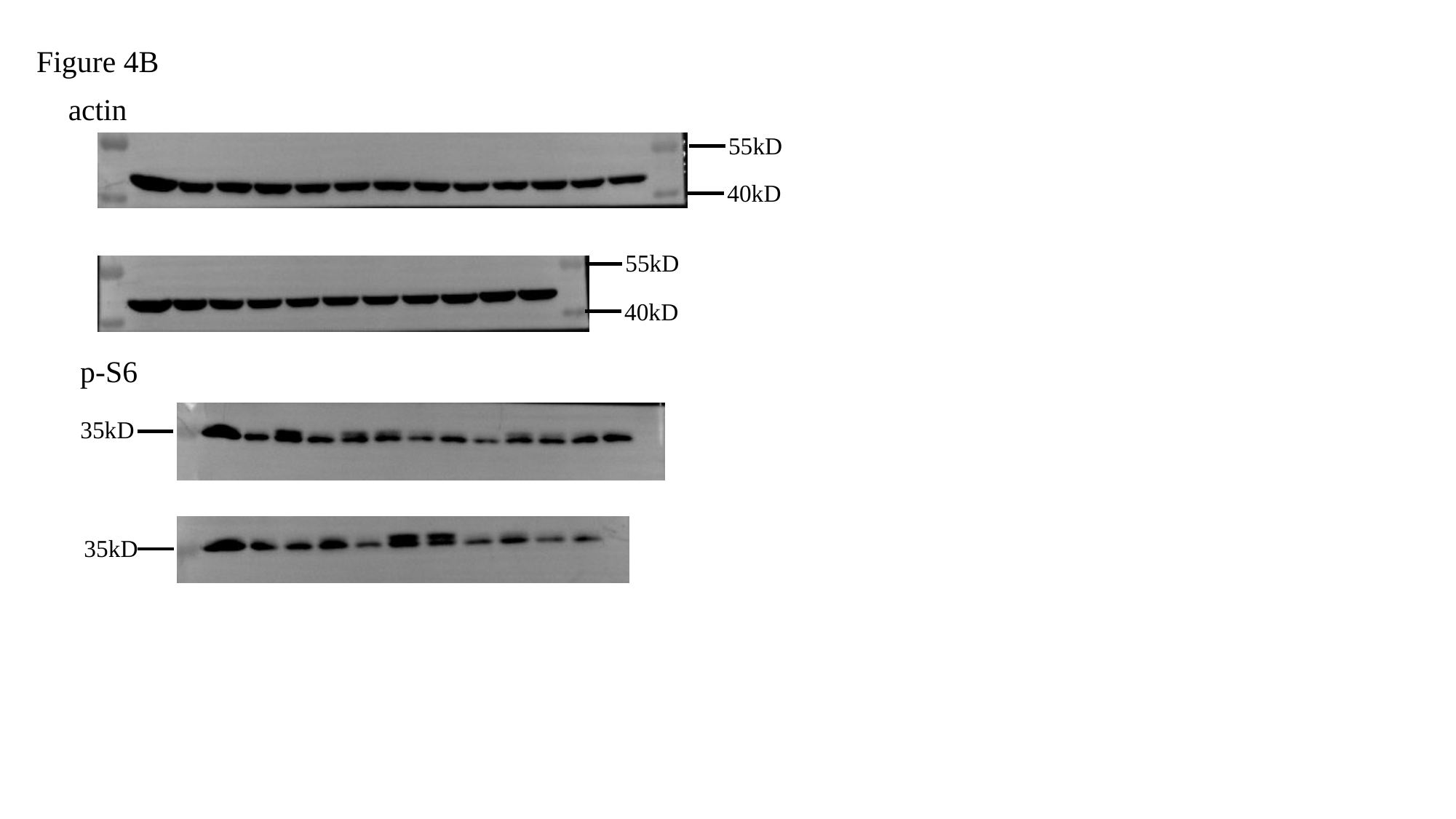

Figure 4B
actin
55kD
40kD
55kD
40kD
p-S6
35kD
35kD

Supplement: Supplementary file 6 — Original Data File [file 41389_2023_499_MOESM6_ESM.pptx]
